# Cardiometabolic index is associated with impaired renal function: Nonlinear threshold effects and dose–response relationships in a national cohort (NHANES 1999–2018)

**DOI:** 10.1097/MD.0000000000043653

**Published:** 2025-08-01

**Authors:** Zhengyang Zhu, Kejun Ren, Xiaowei Duan, Xulei Hu, Yong Lv, Dong Wang, Hua Jin, Lei Zhang

**Affiliations:** a Department of Postgraduate, First Clinical Medical College, Anhui University of Chinese Medicine, Hefei, Anhui Province, China; b Department of Nephrology, First Affiliated Hospital, Anhui University of Chinese Medicine, Hefei, Anhui Province, China.

**Keywords:** cardiometabolic index, chronic kidney disease, estimated glomerular filtration rate, metabolic dysregulation, NHANES, threshold effect

## Abstract

Accurate assessment of renal function is critical for early detection and management of chronic kidney disease (CKD). While estimated glomerular filtration rate (eGFR) remains the gold standard for evaluating renal filtration capacity, its limitations in identifying early metabolic-driven dysfunction necessitate complementary biomarkers. The cardiometabolic index (CMI), reflects metabolic dysregulation but its link to eGFR-defined renal impairment remains unstudied. In this cross-sectional analysis of 13,696 U.S. adults (National Health and Nutrition Examination Survey 1999–2018), eGFR was calculated using the 2021 CKD Epidemiology Collaboration (CKD-EPI) creatinine equation (eGFR < 60 mL/min/1.73m² indicating reduced renal function). CMI, integrating triglyceride-to- high-density-lipoprotein cholesterol ratio (TG/HDL-C) and waist-to-height ratio (WHtR). Weighted multivariable logistic regression models adjusted for sociodemographic, lifestyle, and clinical covariates assessed CMI-eGFR associations. Restricted cubic spline and threshold analyses explored nonlinear relationships, while subgroup analyses tested consistency across populations. Higher CMI quintiles showed a dose-dependent increase in reduced eGFR prevalence (Q1: 5.3% vs Q5: 23.8%, *P* < .001). Fully adjusted models revealed 2.16-fold elevated odds of reduced eGFR in the highest CMI quintile (95% confidence interval (CI): 1.27–3.69) versus the lowest. Restricted cubic spline analysis identified a nonlinear threshold effect at CMI = 0.182: below this threshold, each 1-unit CMI increase raised eGFR risk by 86% (95% CI = 1.49–2.23; *P* = .045), while above it, risk increased by 33% (95% CI = 1.11–1.59; *P* = .002). Subgroup analyses confirmed consistent associations in females, nondiabetics, and older adults (all *P* < .05), with no significant interactions. Elevated CMI independently predicts reduced renal function, underscoring its utility as a metabolic biomarker for early CKD detection. The identified threshold (CMI ≥ 0.182) highlights a critical inflection point for clinical risk stratification, advocating CMI integration into routine screening to guide preventive strategies against metabolic-driven renal impairment.

## 1. Introduction

Chronic kidney disease (CKD), a progressive disorder marked by sustained impairment of renal structure or function (≥3 months), imposes a substantial global health burden, with recent epidemiological data estimating its prevalence in over 700 million individuals.^[1]^ Characterized clinically by diminished eGFR or pathological albuminuria,^[2]^ CKD progression culminates in end-stage renal failure in 10% of cases and accounts for > 2 million preventable deaths annually due to delayed therapeutic intervention.^[3]^ Current diagnostic paradigms prioritize eGFR measurement for renal function assessment, yet emerging evidence underscores the need for complementary biomarkers to detect early-stage metabolic disturbances preceding overt renal decline.^[4]^

The pathophysiology of CKD is increasingly linked to metabolic dysregulation, particularly lipid homeostasis disturbances.^[5]^ Visceral adiposity and insulin resistance – quantifiable through WHtR and TG/HDL-C ratio – have been implicated in renal microvascular dysfunction.^[6]^ CMI, derived from (WHtR × TG)/HDL-C, synthesizes these metabolic and anthropometric parameters into a unified metric.^[7]^ While CMI demonstrates diagnostic utility in cardiovascular and metabolic disorders,^[8]^ its association with renal filtration capacity remains unexplored. This gap is clinically significant given that lipid nephrotoxicity may induce glomerular hypertension and podocyte injury,^[9]^ while insulin resistance alters renal hemodynamics – both mechanisms potentially accelerating eGFR decline.^[10]^ Mechanistic studies suggest that CMI components synergistically contribute to renal pathology^[11]^: WHtR-associated visceral adiposity promotes pro-inflammatory cytokine release,^[12]^ TG-rich lipoproteins induce oxidative stress in renal tubular cells,^[13]^ and low HDL-C impairs endothelial function.^[14]^ These processes may collectively compromise glomerular filtration efficiency, yet conventional lipid markers fail to capture this multidimensional metabolic risk. Notably, CMI’s capacity to integrate central obesity and atherogenic dyslipidemia positions it as potentially associated with subclinical renal impairment, particularly in populations with metabolic syndrome – a key risk factor for CKD development.

Utilizing the NHANES dataset, this cross-sectional analysis investigates the correlation between CMI and eGFR to evaluate its role as a metabolic indicator of renal functional status. By elucidating this relationship, we aim to determine whether CMI could augment current eGFR-based diagnostic frameworks, offering insights into metabolic contributors to early renal dysfunction. This investigation addresses a critical unmet need in nephrology, where early detection of metabolic renal injury remains challenging yet essential for implementing targeted interventions to mitigate CKD progression.

## 2. Methods

### 2.1. Survey overview

This study utilized data from the 1999 to 2018 cycles of the NHANES (https://www.cdc.gov/nchs/nhanes/), administered by the Centers for Disease Control and Prevention (CDC).^[15]^ Following standardized protocols, we systematically collected demographic information through structured interviews while integrating physical examinations, 24-hour dietary recalls, and comprehensive laboratory assessments (including serum biomarker analyses) to evaluate population health metrics.^[16]^ The study protocol received ethical approval from the National Center for Health Statistics Ethics Review Board (compliant with the Declaration of Helsinki principles), with written informed consent obtained from all participants. As a secondary analysis of de-identified public data, no additional ethical review was required.

### 2.2. Study population

**Figure 1.**
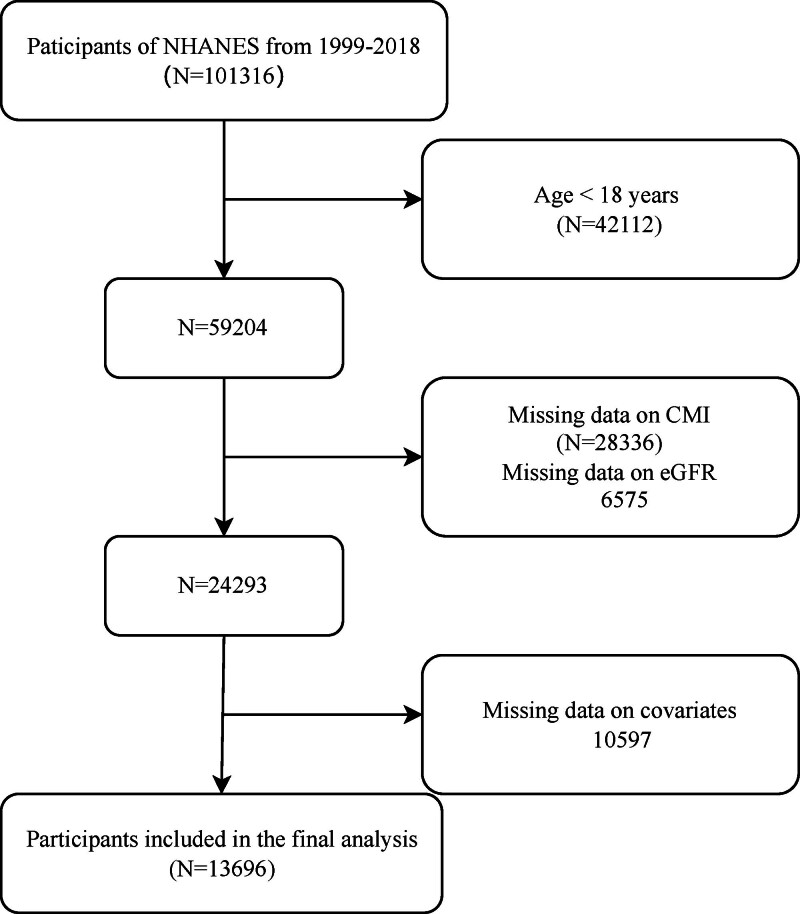
Flowchart of participant selection in NHANES (1999–2018). In the NHANES database, each participant was assigned a unique SEQN identifier. Across different data collection cycles, the same participant’s records were linked and consolidated using their respective SEQN identifiers. NHANES = National Health and Nutrition Examination Survey.

This study utilized data from the National Health and Nutrition Examination Survey (NHANES) spanning 1999 to 2018 to investigate associations between cardiometabolic risk, quantified through CMI, and renal function assessed via eGFR. From an initial pool of 101,316 potentially eligible participants, rigorous exclusion criteria were applied to ensure data quality. The exclusion process proceeded as follows: 6575 individuals were excluded due to missing valid eGFR measurements; 42,112 participants were removed for being under 18 years of age; 27,273 were excluded for incomplete triglyceride measurements required for CMI calculation; and 10,597 were eliminated due to missing essential covariates. Subsequent quality control measures excluded an additional 998 participants with unavailable waist circumference (WC) data and 63 individuals lacking height measurements. Following these sequential exclusions, the final analytic cohort comprised 13,696 adults who met all inclusion criteria and had complete data for the study variables (Fig. 1).

### 2.3. Definition of exposure variable

CMI is an integrated metabolic indicator combining lipid homeostasis and body composition metrics through the mathematical product of 2 components: TG/HDL-C and WHtR.^[17]^ Venous blood specimens were procured after a minimum 9-hour fasting period, with TG and HDL-C analyses exclusively performed on stabilized samples collected in mobile examination centers or standardized clinical facilities to ensure methodological rigor.^[18]^ Anthropometric parameters (WC and standing height) were systematically recorded by trained phlebotomists using validated portable instrumentation. WHtR was defined as WC (cm) divided by height (cm), with CMI calculated as: CMI = (TG (mmol/L)/ HDL-C (mmol/L)) × WHtR. This composite biomarker served as the primary independent variable in our analysis, encapsulating both dyslipidemia patterns and visceral adiposity dimensions within a unified metric.

### 2.4. Definition of outcome variable

Reduced kidney function was defined as an eGFR < 60 mL/min/1.73m², consistent with established criteria for CKD. Blood samples were collected from participants during the physical examination phase. Serum was separated by centrifugation within 2 hours of collection, stored at − 80°C, and subsequently analyzed in a central laboratory certified by the Clinical Laboratory Improvement Amendments. Serum creatinine levels were measured using the enzymatic method traceable to isotope dilution mass spectrometry. eGFR values were calculated using the 2021 CKD-EPI equation (Equation 1),^[19]^ which incorporates serum creatinine, age, sex, and race. All laboratory procedures adhered to standardized protocols outlined in the NHANES Laboratory Operations Manual,^[20]^ with internal quality controls and external proficiency testing conducted biannually to ensure measurement accuracy.


$$\begin{array}{ll} eGFR & =141*min{\left(\frac{Scr}{\kappa },1\right)}^{\alpha }*max{\left(\frac{Scr}{\kappa },1\right)}^{-1.20}\;\\ & *0.993^{Age}*SexFactor*RaceFactor \end{array}$$

**Equation 1:**Scr: Serum creatinine (mg/dL).κ: 0.7 (female), 0.9 (male).α: −0.329 (female), −0.411 (male). Sex factor: 1.018 (female), 1 (male). Race factor: 1.159 (black individuals), 1 (others).

### 2.5. Covariates

This study utilized data collected through standardized protocols from the NHANES. Sociodemographic information was obtained via structured household interviews, while medical evaluations incorporated standardized physical examinations and laboratory testing. Confounding variables were defined based on established research consensus and included the following: age (dichotomized as >60 years vs ≤60 years), sex (male/female), marital status (categorized as married or other (encompassing widowed, divorced, separated, never married, or cohabiting)), race/ethnicity (non-Hispanic White, non-Hispanic Black, Mexican American, or other racial groups), education level (less than high school, high school or equivalent, or college and above), and household income stratified by PIR (poverty-income ratio): low income (PIR ≤ 1.3), middle income (1.3 < PIR ≤ 3.5), and high income (PIR > 3.5). Health-related covariates included body mass index (BMI), calculated from measured weight and height (kg/m²) and categorized as normal weight (<25 kg/m²) or overweight/obesity (≥25 kg/m²) based on standard literature-derived cutoffs, as well as self-reported physician-diagnosed chronic conditions (hypertension and diabetes). Lifestyle variables included physical activity intensity, classified as low physical activity (≥10-minute sessions in the past 30 days inducing mild sweating or slight increases in heart/respiratory rates) or high physical activity (sessions causing substantial sweating or marked elevations in heart/respiratory rates). Smoking status was defined as “no” (applied to individuals who smoked fewer than 100 cigarettes in their lifetime) and “yes” (assigned to those with lifetime consumption of 100 cigarettes or more). Alcohol intake was grouped as “no” (for participants reporting fewer than 12 alcoholic drinks in the prior year) and “yes” (for those consuming 12 or more alcoholic drinks within the same 1-year period). Biochemical parameters included: HDL-C, mg/dL, blood urea nitrogen (BUN, mg/dL), TG, mg/dL, total cholesterol (mg/dL), low-density-lipoprotein cholesterol (LDL-C, mg/dL), and serum creatinine (SCR, mg/dL).

### 2.6. Statistical analysis

Considering the complex survey design of NHANES, all analyses incorporated sample weights, clusters, and strata to comply with recommended analytical guidelines for NHANES data. Participants were categorized into quintiles (Q1–Q5) based on CMI values, with the CMI quintile thresholds defined as follows: Q1: CMI < 0.26; Q2: 0.26 ≤ CMI < 0.41; Q3: 0.41 ≤ CMI < 0.62; Q4: 0.62 ≤ CMI < 1.03; Q5: CMI ≥ 1.03. Continuous variables were summarized using weighted means and standard deviations, while categorical variables were presented as frequencies and percentages. Between-group comparisons of baseline characteristics were performed using weighted one-way ANOVA for continuous variables and Rao-Scott-adjusted Pearson’s chi-square tests for categorical variables, with appropriate application of sample weights in respective analyses. To investigate the association between CMI and eGFR, we calculated odds ratio (OR) with 95% CI using multivariable logistic regression models. Three sequential models were constructed: Model 1 (crude analysis); Model 2 adjusted for sex, age, ethnicity, marital status, PIR, smoking, education level, drinking, and physical activity; and Model 3 further adjusted for BUN, total cholesterol, LDL-C, weight, BMI, hypertension, and diabetes mellitus.^[21]^ Potential nonlinear dose-response relationships between CMI levels and eGFR were explored using multivariable-adjusted restricted cubic spline (RCS) analyses with 4 knots,^[22]^ incorporating covariates from Model 3. Threshold effects were examined through piecewise logistic regression modeling and threshold determination.^[23]^ Subgroup analyses stratified by CMI thresholds were conducted across 8 subgroups (sex, age, BMI, diabetes mellitus, hypertension, physical activity, drinking, and smoking), with interaction effects assessed using likelihood ratio tests. The robustness of observed associations was verified through sensitivity analyses employing multivariable logistic regression and RCS approaches. All analyses were performed in R (version 4.3.2) using the rms (version 8.0-0) and nhanesR (version 1.3) packages. Statistical significance was defined as a 2-tailed *P*-value < .05.

## 3. Results

### 3.1. Characteristics of the study population

As shown in Table 1, This retrospective cohort study included 13,696 participants (49.3% male, 50.7% female), statistically representing 79,297,475 US adults. The cohort demonstrated significant renal function stratification, with 12,792 individuals (93.4% of the study population) classified as having reduced kidney function compared to 904 participants (6.6%) maintaining normal renal parameters. Compared to the normal group, the reduced kidney function group demonstrated significant differences: serum creatinine (0.8 vs 1.5, *P* < .001) and BUN (12.9 vs 22.8, *P* < .001) were elevated, while triglycerides (121.3 vs 135.3, *P* < .001) and LDL-C (117.1 vs 111.3, *P* < .001) were higher, and HDL-C lower (53.9 vs 56.4, *P* < .001). Demographically, this group had a higher proportion of older adults (>60 years: 20.1% vs 87.0%, *P* < .001) and non-Hispanic White individuals (73.4% vs 76.6%, *P* < .001). Socio-behavioral disparities included lower educational attainment (<high school: 14.2% vs 21.1%, *P* < .001), higher smoking rates (46.5% vs 47.6%, *P* < .001), and reduced physical activity (high activity: 67.7% vs 45.0%, *P* < .001). Comorbidities such as hypertension (33.1% vs 80.9%, *P* < .001) and diabetes mellitus (11.8% vs 35.4%, *P* < .001) were significantly more prevalent.

**Table 1:** Basic demographic data of normal kidney function and reduced kidney function subjects.

Variable	Total (n = 13696)	Normal kidney function (n = 904)	Reduced kidney function (n = 12,792)	*P*
BUN (mg/dL)	13.3 (0.1)	22.8 (0.6)	12.9 (0.1)	<.001
SCR (mg/dL)	0.9 (0.0)	1.5 (0.1)	0.8 (0.0)	<.001
TC (mg/dL)	195.3 (0.6)	194.8 (1.7)	195.3 (0.6)	.747
TG (mg/dL)	121.9 (0.9)	135.3 (2.6)	121.3 (0.9)	<.001
LDL-C (mg/dL)	116.9 (0.5)	111.3 (1.4)	117.1 (0.5)	<.001
HDL-C (mg/dL)	54.0 (0.2)	56.4 (0.7)	53.9 (0.2)	<.001
Weight (kg)	81.9 (0.3)	77.0 (0.9)	82.1 (0.3)	<.001
Height (cm)	169.5 (0.1)	163.3 (0.4)	169.8 (0.1)	<.001
WC (cm)	97.6 (0.2)	100.5 (0.7)	97.4 (0.3)	<.001
CMI	0.7 (0.0)	0.8 (0.1)	0.7 (0.0)	<.001
Sex, n (%)				
Male	6769 (49.3)	314 (25.7)	6455 (50.4)	<.001
Female	6927 (50.7)	590 (74.3)	6337 (49.6)	
Ethnicity, n (%)				
Mexican American	2135 (6.9)	60 (2.2)	2075 (7.1)	<.001
Non-Hispanic White	6746 (73.5)	529 (76.6)	6217 (73.3)	
Non-Hispanic Black	2540 (9.1)	227 (14.1)	2313 (8.9)	
Other	2275 (10.5)	88 (7.1)	2187 (10.7)	
Marital status, n (%)				
Married	8452 (66.5)	458 (53.9)	7994 (67.1)	<.001
Other (widowed, divorced, Separated, never married, living with a partner)	5244 (33.5)	446 (46.1)	4798 (32.9)	
PIR, n (%)				
Low income	3883 (18.1)	252 (21.5)	3631 (18.0)	<.001
Middle income	5142 (35.1)	423 (46.1)	4719 (34.6)	
High income	4671 (46.8)	229 (32.4)	4442 (47.4)	
Smoking, n (%)				
No	7416 (53.5)	467 (52.4)	6949 (53.5)	.676
Yes	6280 (46.5)	437 (47.6)	5843 (46.5)	
Education level, n (%)				
Less than high school	3101 (14.5)	265 (21.1)	2836 (14.2)	<.001
High school or equivalent	3080 (22.6)	268 (33.5)	2812 (22.1)	
College or above	7515 (62.9)	371 (45.4)	7144 (63.7)	
Drinking, n (%)				
No	3719 (22.3)	389 (43.9)	3330 (21.4)	<.001
Yes	9977 (77.7)	515 (56.1)	9462 (78.6)	
Physical activity, n (%)				
Low physical activity	5057 (33.3)	534 (55.0)	4523 (32.3)	<.001
High physical activity	8639 (66.7)	370 (45.0)	8269 (67.7)	
Hypertension, n (%)				
No	8177 (64.8)	146 (19.1)	8031 (66.9)	<.001
Yes	5519 (35.2)	758 (80.9)	4761 (33.1)	
Diabetes mellitus, n (%)				
No	11,200 (87.1)	528 (64.6)	10,672 (88.2)	<.001
Yes	2496 (12.9)	376 (35.4)	2120 (11.8)	
Age, n (%)				
≤60	9314 (77.0)	88 (13.0)	9226 (79.9)	<.001
>60	4382 (23.0)	816 (87.0)	3566 (20.1)	
BMI, n (%)				
Normal	4216 (33.0)	247 (29.5)	3969 (33.2)	.091
Overweight	9480 (67.0)	657 (70.5)	8823 (66.8)	

All estimates accounted for complex survey designs.

BMI = body mass index, BUN = blood urea nitrogen, CMI = cardiometabolic index, HDL-C = high-density lipoprotein cholesterol, LDL-C = low-density lipoprotein cholesterol, PIR = poverty-income ratio, SCR = serum creatinine, TC = total cholesterol, TG = triglycerides, WC = waist circumference.

The CMI quintiles demonstrated a graded increase in reduced kidney function prevalence: Q1 (5.3%), Q2 (9.3%), Q3 (14.7%), Q4 (19.5%), and Q5 (23.8%) (*P* < .001 for trend). Higher CMI quintiles correlated with adverse metabolic profiles, including elevated weight (68.2 vs 95.5, *P* < .001), WC (84.6 vs 109.6, *P* < .001), and LDL-C (102.7 vs 122.6, *P* < .001), alongside reduced HDL-C (71.1 vs 39.6, *P* < .001). Renal dysfunction markers worsened progressively, with SCR (0.8 vs 0.9, *P* < .001) and BUN (12.9 vs 13.9, *P* < .001). Demographically, Q5 exhibited higher proportions of older adults (>60 years: 24.7% vs 17.5%, *P* < .001), males (62.4% vs 35.5%, *P* < .001), and non-Hispanic White individuals (75.8% vs 71.9%, *P* < .001). Socioeconomic disparities included lower education (<high school: 18.4% vs 9.3%, *P* < .001) and higher low-income prevalence (20.3% vs 16.6%, *P* = .08). Lifestyle risks, such as smoking (52.5% vs 39.8%, *P* < .001) and physical inactivity (39.5% vs 25.0%, *P* < .001), escalated in higher quintiles. Comorbidities rose markedly, with hypertension (49.0% vs 20.0%, *P* < .001) and diabetes mellitus (24.7% vs 5.1%, *P* < .001) (Table 2).

**Table 2:** Baseline demographic characteristics stratified by CMI quintiles.

Variable	Total (n = 13696)	Q1 (n = 2688)	Q2 (n = 2601)	Q3 (n = 2844)	Q4 (n = 2849)	Q5 (n = 2714)	*P*
BUN (mg/dL)	13.4 (0.1)	12.9 (0.1)	13.1 (0.2)	13.3 (0.1)	13.6 (0.1)	13.9 (0.1)	<.001
SCR (mg/dL)	0.9 (0.0)	0.8 (0.0)	0.9 (0.0)	0.9 (0.0)	0.9 (0.0)	0.9 (0.0)	<.001
TC (mg/dL)	195.3 (0.6)	185.4 (0.9)	189.2 (1.3)	195.8 (1.0)	199.8 (1.1)	206.2 (1.1)	<.001
TG (mg/dL)	122.0 (0.9)	57.9 (0.5)	82.5 (0.6)	107.0 (0.7)	142.1 (1.0)	220.1 (1.9)	<.001
LDL-C (mg/dL)	116.9 (0.5)	102.7 (0.7)	113.1 (1.0)	121.3 (0.8)	124.6 (1.0)	122.6 (1.0)	<.001
HDL-C (mg/dL)	54.0 (0.2)	71.1 (0.4)	59.5 (0.5)	53.2 (0.3)	46.8 (0.3)	39.6 (0.2)	<.001
Weight (kg)	81.9 (0.3)	68.2 (0.4)	76.4 (0.5)	81.4 (0.5)	88.0 (0.6)	95.5 (0.6)	<.001
Height (cm)	169.6 (0.1)	168.5 (0.3)	168.7 (0.3)	169.3 (0.3)	170.1 (0.3)	171.0 (0.3)	<.001
WC (cm)	97.6 (0.2)	84.6 (0.3)	92.5 (0.4)	97.6 (0.4)	103.4 (0.4)	109.6 (0.5)	<.001
Sex, n (%)							
Male	6769 (49.3)	1015 (35.5)	1142 (41.7)	1421 (49.5)	1545 (57.3)	1646 (62.4)	<.001
Female	6927 (50.7)	1673 (64.5)	1459 (58.3)	1423 (50.5)	1304 (42.7)	1068 (37.6)	
Ethnicity, n (%)							
Mexican American	2135 (6.9)	265 (4.9)	294 (5.1)	482 (7.7)	520 (8.1)	574 (8.5)	<.001
Non-Hispanic White	6746 (73.5)	1245 (71.9)	1306 (74.1)	1350 (72.9)	1400 (72.8)	1445 (75.8)	
Non-Hispanic Black	2540 (9.1)	735 (13.9)	571 (10.2)	545 (9.3)	434 (7.4)	255 (4.9)	
Other	2275 (10.5)	443 (9.3)	430 (10.6)	467 (10.1)	495 (11.7)	440 (10.8)	
Marital status, n (%)							
Married	8452 (66.5)	1475 (62.2)	1577 (65.2)	1775 (66.7)	1833 (68.9)	1792 (69.7)	<.001
Other (widowed, divorced, Separated, never married, living with a partner)	5244 (33.5)	1213 (37.8)	1024 (34.8)	1069 (33.3)	1016 (31.1)	922 (30.3)	
PIR, n (%)							
Low income	3883 (18.1)	687 (16.6)	688 (17.3)	783 (17.9)	827 (18.6)	898 (20.3)	<.001
Middle income	5142 (35.1)	966 (32.3)	959 (34.6)	1087 (34.9)	1118 (37.4)	1012 (36.2)	
High income	4671 (46.8)	1035 (51.1)	954 (48.1)	974 (47.2)	904 (44.0)	804 (43.5)	
Smoking, n (%)							
No	7416 (53.5)	1685 (60.3)	1464 (56.9)	1556 (52.4)	1438 (50.2)	1273 (47.5)	<.001
Yes	6280 (46.5)	1003 (39.7)	1137 (43.1)	1288 (47.6)	1411 (49.8)	1441 (52.5)	
Education level, n (%)							
Less than high school	3101 (14.5)	414 (9.2)	510 (13.0)	687 (15.6)	698 (16.3)	792 (18.4)	<.001
High school or equivalent	3080 (22.6)	511 (18.4)	557 (22.1)	637 (22.3)	706 (24.3)	669 (26.0)	
College or above	7515 (62.9)	1763 (72.4)	1534 (64.9)	1520 (62.1)	1445 (59.4)	1253 (55.6)	
Drinking, n (%)							
No	3719 (22.4)	670 (19.1)	702 (22.1)	789 (22.7)	823 (24.4)	735 (23.4)	.008
Yes	9977 (77.6)	2018 (80.9)	1899 (77.9)	2055 (77.3)	2026 (75.6)	1979 (76.6)	
Physical activity, n (%)							
Low physical activity	5057 (33.3)	763 (25.0)	920 (32.4)	1065 (33.8)	1157 (36.0)	1152 (39.5)	<.001
High physical activity	8639 (66.7)	1925 (75.0)	1681 (67.6)	1779 (66.2)	1692 (64.0)	1562 (60.5)	
Hypertension, n (%)							
No	8177 (64.8)	2003 (80.0)	1678 (71.6)	1658 (63.7)	1523 (57.7)	1315 (51.0)	<.001
Yes	5519 (35.2)	685 (20.0)	923 (28.4)	1186 (36.3)	1326 (42.3)	1399 (49.0)	
Diabetes mellitus, n (%)							
No	11,200 (87.2)	2490 (94.9)	2307 (93.4)	2344 (88.1)	2189 (84.1)	1870 (75.3)	<.001
Yes	2496 (12.8)	198 (5.1)	294 (6.6)	500 (11.9)	660 (15.9)	844 (24.7)	
EGFR, n (%)							
Normal	904 (4.3)	114 (2.8)	167 (3.8)	190 (4.2)	219 (5.5)	214 (5.3)	<.001
Reduced kidney function	12,792 (95.7)	2574 (97.2)	2434 (96.2)	2654 (95.8)	2630 (94.5)	2500 (94.7)	
Age, n (%)							
≤60	9314 (77.0)	2064 (82.5)	1814 (78.7)	1854 (75.2)	1808 (73.4)	1774 (75.3)	<.001
>60	4382 (23.0)	624 (17.5)	787 (21.3)	990 (24.8)	1041 (26.6)	940 (24.7)	
BMI, n (%)							
Normal	4216 (33.0)	1705 (67.0)	1055 (44.7)	769 (29.0)	447 (16.4)	240 (7.9)	<.001
Overweight	9480 (67.0)	983 (33.0)	1546 (55.3)	2075 (71.0)	2402 (83.6)	2474 (92.1)	

All estimates accounted for complex survey designs.

BMI = body mass index, BUN = blood urea nitrogen, CMI = cardiometabolic index, EGFR = estimated glomerular filtration rate, HDL-C = high-density lipoprotein cholesterol, LDL-C = low-density lipoprotein cholesterol, PIR = poverty-income ratio, Q1–Q5 = quintile 1 to quintile 5, SCR = serum creatinine, SD = standard deviation, TC = total cholesterol, TG = triglycerides, WC = waist circumference.

### 3.2. The correlation between CMI and CKD incidence

As shown in Table 3, CMI exhibited a consistent association with the outcome across sequential regression models. The unadjusted Model 1 demonstrated an OR of 1.23 **(a 23% increased risk per 1.0-unit increase**) (95% confidence interval [CI]: 1.12–1.36, *P* < .01), which persisted with minimal attenuation in the fully adjusted Model 3 (OR = 1.28 **(a 28% increased risk per 1.0-unit increase**), 95% CI = 1.03–1.58, *P* = .26). A dose-response relationship was evident in the CMI quintile analysis, with progressively elevated risks from Q2 to Q5 relative to the reference quintile (Q1). Sensitivity analyses using CMI quintiles demonstrated stable risk trends across models, particularly for the highest quintile (Q5), supporting the robustness of the observed associations. In Model 1, the ORs for Q2, Q3, Q4, and Q5 were 1.37 (95% CI = 0.98–1.91, *P* = .67), 1.51 (95% CI = 1.07–2.13, *P* = .22), 1.98 (95% CI = 1.39–2.83, *P* < .01), and 1.91 (95% CI = 1.43–2.56, *P* < .01), respectively. In Model 3, the highest quintiles (Q4: OR = 1.76, 95% CI = 1.04–2.97, *P* = .37; Q5: OR = 2.16, 95% CI = 1.27–3.69, *P* = .05) maintained elevated risk trends, though statistical significance was not observed for intermediate quintiles (Q2 and Q3). Notably, the highest CMI quintile (Q5) exhibited a 116% increased risk compared to the reference group in the fully adjusted model (Model 3: OR = 2.16), underscoring a robust association between elevated CMI levels and the outcome.

**Table 3:** Association between CMI and eGFR in patients of NHANES.

Variables	Model1*		Model2†		Model3‡	
	OR (95% CI)	*P*	OR (95% CI)	*P*	OR (95% CI)	*P*
CMI	1.23 (1.12–1.36)	<.001	1.35 (1.16–1.57)	<.001	1.28 (1.03–1.58)	.026
CMI quintile						
Q1	1.00 (Reference)	–	1.00 (Reference)	–	1.00 (Reference)	–
Q2	1.37 (0.98–1.91)	.067	1.07 (0.71–1.60)	.761	1.15 (0.68–1.96)	.599
Q3	1.51 (1.07–2.13)	.022	1.10 (0.74–1.65)	.631	1.28 (0.78–2.11)	.333
Q4	1.98 (1.39–2.83)	<.001	1.56 (1.02–2.38)	.044	1.76 (1.04–2.97)	.037
Q5	1.91 (1.43–2.56)	<.001	1.85 (1.28–2.69)	.001	2.16 (1.27–3.69)	.005

BMI = body mass index, BUN = blood urea nitrogen, CI = confidence interval, CMI = cardiometabolic index, eGFR = estimated glomerular filtration rate, LDL-C = low-density lipoprotein cholesterol, NHANES = National Health and Nutrition Examination Survey, OR = odds ratio, PIR = poverty-to-income ratio, TC = Total cholesterol.

* Model 1: crude analysis.

† Model 2: adjusted for sex, age, ethnicity, marital status, PIR, smoking status, educational attainment, alcohol consumption, and physical activity.

‡ Model 3: adjusted for Model 2 covariates + BUN, TC, LDL-C, body weight, BMI, hypertension, and diabetes mellitus.

### 3.3. Nonlinear associations and threshold analysis

**Figure 2.**
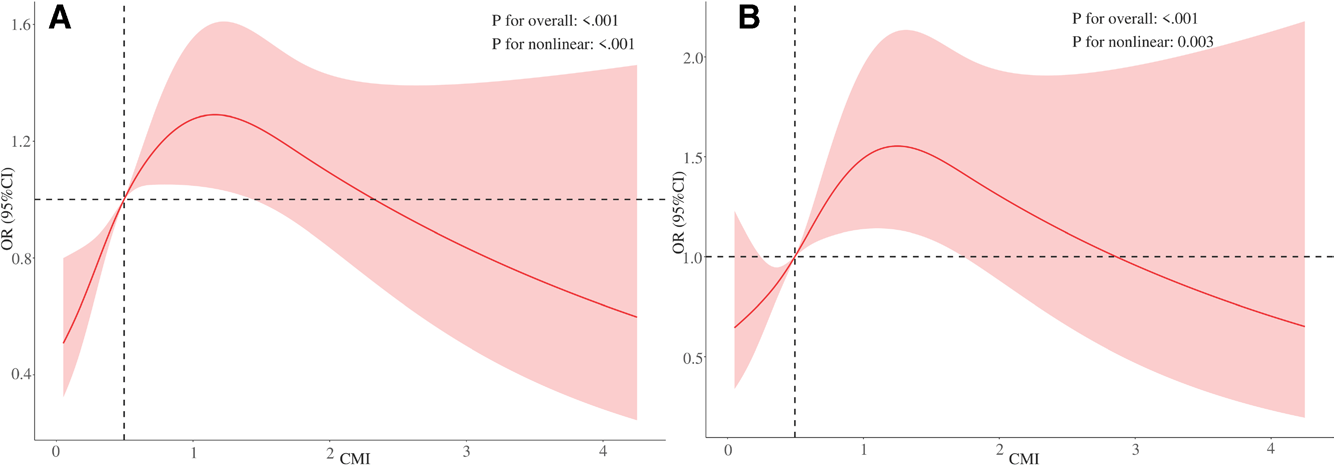
The nonlinear association between CMI and eGFR. (A) Unadjusted RCS and (B) Adjusted RCS with comprehensive adjustments for sex, age, ethnicity, marital status, PIR, smoking status, educational attainment, alcohol consumption, physical activity level, BUN, TC, LDL-C, body weight, BMI, hypertension, and diabetes mellitus. BMI = body mass index, BUN = blood urea nitrogen, CMI = cardiometabolic index, CI = confidence interval, eGFR = estimated glomerular filtration rate, LDL-C, low-density lipoprotein cholesterol, OR = odds ratio, RCS = restricted cubic spline, TC = total cholesterol.

RCS analysis demonstrated a significant inverted U-shaped association between CMI and eGFR (*P* for overall association < .001; *P* for non-linearity = .003) (Fig. 2). In threshold effect analyses, a 1-unit increase in CMI was associated with a 1.86-fold elevation in eGFR (95% CI 1.49–2.23, *P* = .045) when CMI levels were below 0.182. Conversely, in subjects with CMI ≥ 0.182, each 1-unit increment in CMI corresponded to a 1.33-fold increase in eGFR (95% CI 1.11–1.59, *P* = .002), with significant threshold heterogeneity confirmed by likelihood ratio test (*P* = .008) (Table 4).

**Table 4:** Association between CMI and eGFR using 2-piecewise regression models.

CMI	Adjusted Model* 95%CI	*P*
<0.182	1.86 (1.49–2.23)	.045
≥0.182	1.33 (1.11–1.59)	.002
*P* for likelihood test		.008

BMI = body mass index, BUN = blood urea nitrogen, CI = confidence interval, CMI = cardiometabolic index, eGFR = estimated glomerular filtration rate, LDL-C = low-density lipoprotein cholesterol, OR = odds ratio, PIR = poverty-to-income ratio, TC = total cholesterol.

* Adjusted for sex, age, ethnicity, marital status, PIR, smoking status, educational attainment, alcohol consumption, physical activity, BUN, TC, LDL-C, body weight, BMI, hypertension, and diabetes mellitus.

### 3.4. Subgroup analyses

**Figure 3.**
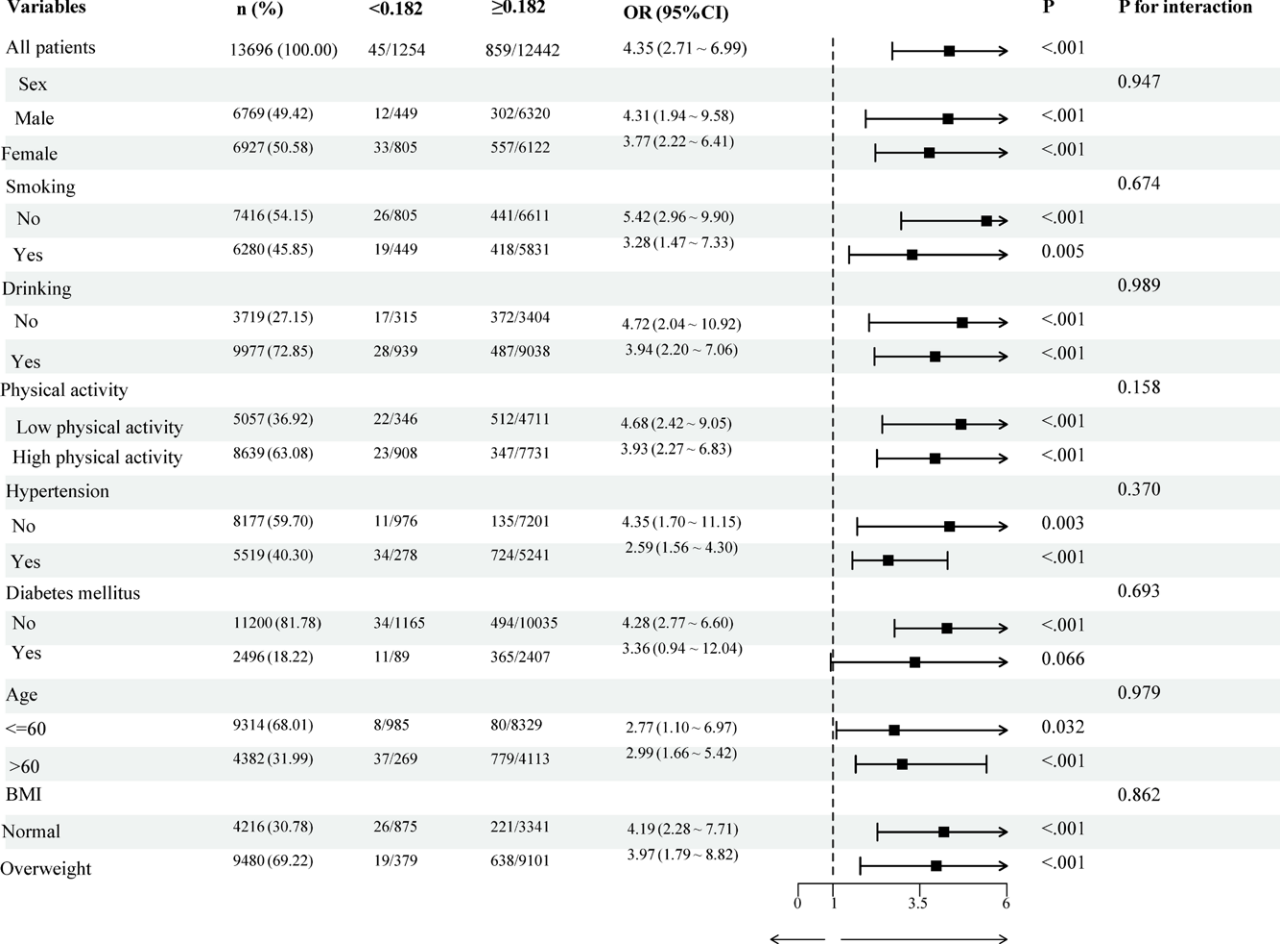
Subgroup analyses were conducted to investigate the association between CMI and eGFR, with mutual adjustments for ethnicity, marital status, PIR, education level, BUN, TC, LDL-C, and weight. BMI = body mass index, BUN = blood urea nitrogen, CMI = cardiometabolic index, CI = confidence interval, eGFR, estimated glomerular filtration rate, LDL-C, low-density lipoprotein cholesterol, OR = odds ratio, PIR = poverty-to-income ratio, TC = total cholesterol.

Subgroup analyses were conducted to investigate the potential effects of CMI on eGFR across diverse populations. As illustrated in Figure 3, significant associations between CMI and eGFR were observed in the following subgroups: females (OR = 3.77, 95% CI = 2.22–6.41; *P* < .001), nonsmokers (OR = 5.42, 95% CI = 2.96–9.90; *P* < .001), alcohol consumers (OR = 3.94, 95% CI = 2.20–7.06; *P* < .001), individuals with low physical activity (OR = 4.68, 95% CI = 2.42–9.05; *P* < .001), non-hypertensive patients (OR = 4.35, 95% CI = 1.70–11.15; *P* = .003), nondiabetic individuals (OR = 4.28, 95% CI = 2.77–6.60; *P* < .001), participants aged > 60 years (OR = 2.99, 95% CI = 1.66–5.42; *P* < .001), and those with normal BMI (OR = 4.19, 95% CI = 2.28–7.71; *P* < .001) or overweight (OR = 3.97, 95% CI = 1.79–8.82; *P* < .001). These associations remained robust after full adjustment for all covariates. Notably, interaction tests revealed no significant effect modification by any of the examined variables (all interaction *P* > .05), indicating homogeneity in the CMI-eGFR relationship across subgroups.

## 4. Discussion

CMI, a composite metric integrating TG/HDL-C and WHtR, has emerged as a novel biomarker for metabolic dysregulation.^[24]^ In this large-scale, nationally representative study of 13,696 U.S. adults, we systematically evaluated its association with renal functional decline, quantified by eGFR. Our findings reveal a robust, dose-dependent relationship between ascending CMI levels and deteriorating renal function,^[25]^ independent of traditional risk factors. Participants in the highest CMI quintile exhibited 2.16-fold greater odds of reduced eGFR (95% CI = 1.27–3.69; *P* = .05) compared to the lowest quintile after comprehensive adjustment for sociodemographic, lifestyle, and clinical confounders. This graded association persisted across diverse demographic strata, underscoring CMI’s capacity to encapsulate multifaceted metabolic insults contributing to subclinical nephropathy.^[26]^

The pathophysiological plausibility of these observations lies in CMI’s dual capacity to reflect atherogenic dyslipidemia and visceral adiposity^[27]^ – two interrelated pathways implicated in renal injury. Elevated TG/HDL-C ratios, indicative of lipid homeostasis disruption, promote renal tubular oxidative stress through free fatty acid-mediated mitochondrial dysfunction^[28]^ and endoplasmic reticulum stress. Experimental models demonstrate that free fatty acids overload proximal tubule cells, impairing fatty acid β-oxidation^[29]^ and generating reactive oxygen species, which trigger apoptosis and interstitial fibrosis. Concurrently, WHtR-quantified central adiposity exacerbates glomerular hypertension via pro-inflammatory cytokine cascades^[30]^ (e.g., IL-6, TNF-α) and adipokine imbalances (reduced adiponectin, elevated leptin). Visceral adipose tissue, metabolically active and hypoxia-prone, secretes chemokines that recruit macrophages, perpetuating a state of chronic low-grade inflammation. This inflammatory milieu directly damages podocytes and mesangial cells, accelerating glomerulosclerosis – a hallmark of progressive CKD.^[31]^

Threshold analysis further identified a critical inflection point at CMI = 0.182 (*P* for interaction = 0.008),^[32]^ beyond which each unit increase in CMI amplified renal risk by 33% (95% CI = 1.11–1.59). This nonlinear trajectory aligns with experimental evidence of cumulative renal insults from synergistic lipidotoxicity^[33]^ and ectopic fat deposition. Beyond this threshold, adipose tissue expandability may reach its limit, leading to ectopic lipid accumulation in renal parenchyma – a phenomenon termed “lipotoxicity.” Lipid-laden renal cells exhibit impaired autophagy and increased apoptosis, exacerbating tubular atrophy and interstitial fibrosis. Such threshold effects highlight the clinical relevance of early intervention to prevent metabolic overload in high-risk populations.

CMI’s superiority to isolated anthropometric indices (e.g., BMI, WC) stems from its integration of lipid derangements with fat distribution patterns. While BMI conflates muscle mass and adiposity, and WC fails to distinguish subcutaneous from visceral fat, CMI’s composite architecture aligns with contemporary paradigms emphasizing dysfunctional adipose signaling over mere adipocyte expansion. For instance, in the Look AHEAD trial, BMI and WC showed moderate associations with albuminuria (OR = 1.72 and 1.75, respectively), yet these metrics lack specificity for visceral adiposity-driven metabolic dysregulation. In contrast, CMI’s incorporation of TG/HDL-C ratio – a marker of insulin resistance and atherogenic lipoprotein profiles – provides a more holistic assessment of metabolic health. This advantage is particularly evident in normoglycemic individuals, where CMI identified significant renal risk (OR = 4.28, 95% CI = 2.77–6.60) despite the absence of overt diabetes, suggesting its utility in detecting subclinical metabolic insults preceding hyperglycemia.

Subgroup analyses revealed pronounced susceptibility in specific cohorts: sex-specific vulnerability: females exhibited 3.77-fold elevated odds (95% CI = 2.22–6.41; *P* < .001), potentially attributable to estrogen-modulated lipid metabolism. Estrogen deficiency in postmenopausal women enhances visceral adiposity and reduces HDL-C, exacerbating renal lipotoxicity. Aging-related susceptibility: adults > 60 years demonstrated 2.99-fold risk escalation (95% CI = 1.66–5.42; *P* < .001), consistent with age-associated declines in renal reserve, mitochondrial biogenesis, and antioxidant defenses. Metabolic paradox in nondiabetics: The stronger association in nondiabetic individuals (OR = 4.28 vs diabetics; *P* < .001) may reflect compensatory hyperfiltration masking early renal damage in diabetics, whereas CMI detects preclinical metabolic dysregulation in normoglycemic subjects.

These findings advocate for CMI’s integration into primary care risk stratification protocols.^[34]^ The identified threshold (CMI ≥ 0.182) could guide personalized interventions – structured weight loss,^[35]^ Mediterranean diets, or PPAR-γ agonists – to mitigate visceral adiposity and dyslipidemia. For instance, a 5% reduction in WHtR through lifestyle modification may lower CMI below the risk threshold, potentially attenuating renal decline.

Despite methodological rigor from NHANES’s standardized protocols and complex survey weighting, several limitations warrant consideration. First, the cross-sectional design precludes causal inference; prospective cohort studies are needed to validate CMI’s predictive utility for incident CKD. Second, while we adjusted for major confounders, residual confounding from unmeasured variables (e.g., dietary sodium intake, gut microbiota profiles, APOL1 genetic variants) may influence observed associations. Third, reliance on single-timepoint biomarker measurements introduces potential misclassification bias, as metabolic parameters fluctuate over time.

Future research should prioritize 3 avenues: longitudinal validation: track CMI trajectories in relation to hard endpoints (e.g., dialysis initiation, cardiovascular events) across diverse populations. Mechanistic elucidation: employ multi-omics approaches to delineate molecular pathways linking adipose-renal crosstalk, particularly the role of extracellular vesicles carrying adipocyte-derived miRNAs. Interventional trials: Evaluate whether CMI-guided therapies (e.g., GLP-1 agonists targeting visceral fat) delay CKD progression in high-risk cohorts.

## 5. Conclusion

Elevated CMI independently correlates with diminished renal function, underscoring its role as a **strongly associated indicator** of metabolic-driven kidney impairment. The identification of a specific CMI threshold highlights its potential for early risk stratification in clinical settings. Integrating CMI into routine assessments could enhance personalized interventions, while further longitudinal research is warranted to confirm its prognostic relevance in CKD progression.

## Acknowledgments

We would like to acknowledge the participants and investigators of the National Health and Nutrition Examination Survey. The content is solely the responsibility of the authors and does not necessarily represent the official views of the National Institutes of Health.

## Author contributions

**Data curation:** Zhengyang Zhu.

**Formal analysis:** Zhengyang Zhu, Xiaowei Duan.

**Investigation:** Kejun Ren, Xulei Hu.

**Methodology:** Zhengyang Zhu.

**Resources:** Kejun Ren, Hua Jin.

**Supervision:** Hua Jin.

**Visualization:** Zhengyang Zhu.

**Writing – original draft:** Zhengyang Zhu, Kejun Ren, Lei Zhang.

**Writing – review & editing:** Xiaowei Duan, Xulei Hu, Yong Lv, Dong Wang, Hua Jin, Lei Zhang.
